# Burden of enteric fever at three urban sites in Africa and Asia: a multicentre population-based study

**DOI:** 10.1016/S2214-109X(21)00370-3

**Published:** 2021-11-16

**Authors:** James E Meiring, Mila Shakya, Farhana Khanam, Merryn Voysey, Maile T Phillips, Susan Tonks, Deus Thindwa, Thomas C Darton, Sabina Dongol, Abilasha Karkey, K Zaman, Stephen Baker, Christiane Dolecek, Sarah J Dunstan, Gordon Dougan, Kathryn E Holt, Robert S Heyderman, Firdausi Qadri, Virginia E Pitzer, Buddha Basnyat, Melita A Gordon, John Clemens, Andrew J Pollard, STRATAA Study Group

**Affiliations:** a Oxford Vaccine Group, Department of Paediatrics, University of Oxford, and the NIHR Oxford Biomedical Research Centre, Oxford, UK; b Malawi-Liverpool-Wellcome Trust Clinical Research Programme, Blantyre, Malawi; c Oxford University Clinical Research Unit, Patan Academy of Health Sciences, Kathmandu, Nepal; d Patan Academy of Health Sciences, Patan Hospital, Lalitpur, Nepal; e International Centre for Diarrhoeal Diseases Research, Bangladesh, Dhaka, Bangladesh; f Department of Epidemiology of Microbial Diseases, Yale School of Public Health, Yale University, New Haven, CT, USA; g College of Medicine, University of Malawi, Blantyre, Malawi; h Department of Infection, Immunity and Cardiovascular Disease, University of Sheffield, Sheffield, UK; i Department of Medicine, University of Cambridge, Cambridge, UK; j Nuffield Department of Medicine, Centre for Tropical Medicine and Global Health, University of Oxford, Oxford, UK; k Mahidol Oxford Tropical Medicine Research Unit, Mahidol University, Bangkok, Thailand; l The Peter Doherty Institute for Infection and Immunity, The University of Melbourne, Melbourne, VIC, Australia; m Department of Infectious Diseases, Central Clinical School, Monash University, Melbourne, VIC, Australia; n Department of Infection Biology, London School of Hygiene & Tropical Medicine, London, UK; o National Institute for Health Research Global Health Research Unit on Mucosal Pathogens, Division of Infection and Immunity, University College London, London, UK; p Institute of Infection, Veterinary and Ecological Sciences, University of Liverpool, Liverpool, UK

## Abstract

**Background:**

Enteric fever is a serious public health concern in many low-income and middle-income countries. Numerous data gaps exist concerning the epidemiology of *Salmonella enterica* serotype Typhi (*S* Typhi) and *Salmonella enterica* serotype Paratyphi (*S* Paratyphi), which are the causative agents of enteric fever. We aimed to determine the burden of enteric fever in three urban sites in Africa and Asia.

**Methods:**

In this multicentre population-based study, we did a demographic census at three urban sites in Africa (Blantyre, Malawi) and Asia (Kathmandu, Nepal and Dhaka, Bangladesh) between June 1, 2016, and Sept 25, 2018. Households were selected randomly from the demographic census. Participants from within the geographical census area presenting to study health-care facilities were approached for recruitment if they had a history of fever for 72 h or more (later changed to >48 h) or temperature of 38·0°C or higher. Facility-based passive surveillance was done between Nov 11, 2016, and Dec 31, 2018, with blood-culture collection for febrile illness. We also did a community-based serological survey to obtain data on Vi-antibody defined infections. We calculated crude incidence for blood-culture-confirmed *S* Typhi and *S* Paratyphi infection, and calculated adjusted incidence and seroincidence of *S* Typhi blood-culture-confirmed infection.

**Findings:**

423 618 individuals were included in the demographic census, contributing 626 219 person-years of observation for febrile illness surveillance. 624 *S* Typhi and 108 *S* Paratyphi A isolates were collected from the blood of 12 082 febrile patients. Multidrug resistance was observed in 44% *S* Typhi isolates and fluoroquinolone resistance in 61% of *S* Typhi isolates. In Blantyre, the overall crude incidence of blood-culture confirmed *S* Typhi was 58 cases per 100 000 person-years of observation (95% CI 48–70); the adjusted incidence was 444 cases per 100 000 person-years of observation (95% credible interval [CrI] 347–717). The corresponding rates were 74 (95% CI 62–87) and 1062 (95% CrI 683–1839) in Kathmandu, and 161 (95% CI 145–179) and 1135 (95% CrI 898–1480) in Dhaka. *S* Paratyphi was not found in Blantyre; overall crude incidence of blood-culture-confirmed *S* Paratyphi A infection was 6 cases per 100 000 person-years of observation (95% CI 3–11) in Kathmandu and 42 (95% CI 34–52) in Dhaka. Seroconversion rates for *S* Typhi infection per 100 000 person-years estimated from anti-Vi seroconversion episodes in serological surveillance were 2505 episodes (95% CI 1605–3727) in Blantyre, 7631 (95% CI 5913–9691) in Kathmandu, and 3256 (95% CI 2432–4270) in Dhaka.

**Interpretation:**

High disease incidence and rates of antimicrobial resistance were observed across three different transmission settings and thus necessitate multiple intervention strategies to achieve global control of these pathogens.

**Funding:**

Wellcome Trust and the Bill & Melinda Gates Foundation.

## Introduction

Enteric fever is estimated to cause 11–18 million infections and 100 000–200 000 deaths globally each year, resulting in a considerable public health burden in many low-income and middle-income countries in Africa and Asia.^1^, ^2^ The human-restricted pathogens *Salmonella enterica* serovars Typhi (*S* Typhi) and Paratyphi A, B, and C cause enteric fever, which presents as a non-specific febrile illness after the oral ingestion of contaminated food or water, with a reported case fatality rate of around 2·5% despite antimicrobial treatment.^3^ High rates of disease have consistently been reported at urban sites in Asia,^4^ with an increasing proportion of *S* Paratyphi A in some sites.^5^ In Africa, the cross-continental introduction of the H58 pathovar from Asia has coincided with a documented increase in cases of *S* Typhi, often associated with large outbreaks, persisting for years, at multiple African sites^6^ and a concerning increase in antimicrobial resistance.^7^, ^8^ The age profile for disease burden differs across different epidemiological contexts.^4^

Various factors lead to underestimation of the true incidence of disease, including the non-specific presentation of the disease,^9^ the absence of accurate point-of-care diagnostics,^10^ and the often indiscriminate empirical use of antimicrobials for fever syndromes without a specific diagnosis in endemic countries.^11^

**Research in context**

**Evidence before this study**

We considered evidence from a systematic review of typhoid fever incidence studies published in February, 2017, in addition to a PubMed search for articles done on Jan 1, 2021, using the search terms “(typhoid OR Salmonella Typhi) AND seroincidence”. Our search yielded 33 studies from sites in 21 countries that had reported on the incidence of blood-culture-confirmed typhoid fever. No population-based incidence studies of enteric fever have been done in Malawi or Nepal. Two previous studies done in Dhaka, Bangladesh reported typhoid fever incidence estimates of 395 cases per 100 000 person-years for the period 2000–01 and 280 cases per 100 000 person-years for the period 2003–04. No previous studies have estimated the seroincidence of infection with *Salmonella enterica* serotype Typhi (*S* Typhi).

**Added value of this study**

We provide the first population-based enteric fever incidence estimates for Blantyre, Malawi and Kathmandu, Nepal, and updated estimates of incidence in Dhaka, Bangladesh. This is also the first study to compare population-based estimates of typhoid fever incidence with estimates of the seroincidence of *S* Typhi infection on the basis of serial anti-Vi IgG titres. We found a high incidence of typhoid fever, with overall crude and adjusted incidence of blood-culture-confirmed *S* Typhi per 100 000 person-years of observation of 58 cases (95% CI 48–70) and 477 cases (95% credible interval [CrI] 372–770) in Blantyre, 74 (95% CI 62–87) and 1065 (95% CrI 687–1824) in Kathmandu, and 161 (95% CI 145–179) and 1138 (95% CrI 889–1477) in Dhaka, respectively. Seroconversion rates for *S* Typhi infection per 100 000 person-years estimated from anti-Vi seroconversion episodes in serological surveillance were 2505 (95% CI 1605–3727) in Blantyre, 7631 (95% CI 5913–9691) in Kathmandu, and 3256 (95% CI 2432–4270) in Dhaka. Additionally, we found high rates of antimicrobial resistance across the three sites, with multidrug resistance identified in 44% of isolates and fluoroquinolone resistance identified in 61% of isolates.

**Implications of all the available evidence**

The high burden of typhoid fever at these study sites provides evidence to support decision making on typhoid conjugate vaccine introduction in these countries. Paratyphoid fever is rarely observed in sub-Saharan Africa, but accounts for 5–25% of enteric fever cases in south Asia. Antimicrobial resistance is common among isolates of both *S* Typhi and *S enterica* serotype Paratyphi A, but patterns of resistance vary between sub-Saharan Africa and south Asia. Passive surveillance of blood-culture-confirmed enteric fever cases considerably underestimate the true incidence of the disease. Seroincidence data can complement traditional population-based surveillance of blood-culture-confirmed typhoid fever, but further research is needed to interpret the significance of increases in anti-Vi titres.

Using data gaps identified through a typhoid transmission model,^12^ the Strategic Typhoid Alliance across Africa and Asia (STRATAA) consortium designed a comprehensive study at three sites combining household-level and individual-level demographic census, health-care utilisation surveys, and enhanced facility-based passive surveillance. Community-based serological surveys were also done to further characterise undiagnosed exposure to the bacteria. Here, we present the overall and adjusted incidence of blood-culture-confirmed *S* Typhi and *S* Paratyphi at three urban sites in Africa (Blantyre, Malawi) and Asia (Kathmandu, Nepal and Dhaka, Bangladesh) and seroconversion rates for *S* Typhi.

## Methods

### Study design and site selection

**Figure 1:**
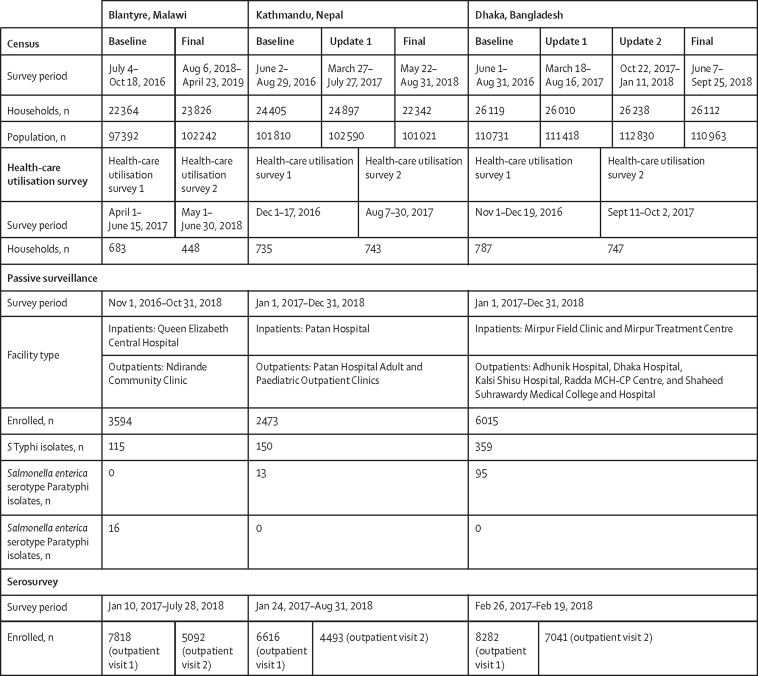
Dates and recruitment numbers for each component of the study

The STRATAA programme is a prospective observational, population-based collaborative and multidisciplinary epidemiological study, comprising health-facility-based passive surveillance for febrile illness, community-based serological surveys, and health-care utilisation surveys nested within a demographic household census (figure 1). A detailed description of the study sites, design, and methods (including sample size considerations) has been previously published.^13^

Briefly, the study was done in three urban areas within the Ndirande township of Blantyre, Malawi, the Lalitpur area of Kathmandu, Nepal, and Mirpur, Dhaka, Bangladesh. Further information on the study sites is provided in the appendix (p 1). Individuals who were resident within households inside the demarcated geographical area were eligible for inclusion in the demographic census, with a household defined as individuals living in the same dwelling or compound and sharing food from the same kitchen. Approval for the joint study protocol was obtained from local research ethics committees at all participating centres and the Oxford Tropical Research Ethics committee.

### Demographic census, census update, and health-care utilisation surveys

Within a demarcated geographical area, a baseline census population was enumerated over a 3–5-month period (June 1–Oct 18, 2016). Consent and demographic information were collected from the head of the household. Each household and individual were given a unique household and member identification number and global positioning system (GPS) coordinates were recorded. A census update was done three times in Dhaka at intervals of 6 months and once in Kathmandu at 1 year, and all three sites had a final census at 2 years. All data were collected through Open Data Kit Collect using Android-based tablet devices, and stored on a MySQL database.^14^

Two health-care utilisation surveys were done at each site between 2016 and 2018. Approximately 735 households with children were interviewed per survey to estimate the probability that those with fever would seek health care at a designated study facility (appendix p 6). Households were selected randomly from the demographic census.

### Passive surveillance

Participants within the demographic census area were encouraged to attend study health-care facilities for blood culture and treatment if they developed fever. Participants presenting from within the geographical census area were approached for recruitment if they had a history of fever for 72 h or more (later changed to ≥48 h) or current temperature of 38·0°C or higher (appendix p 1).

### Serosurveys

Approximately 8500 participants were randomly selected in an age-stratified manner from the original demographic census (appendix p 7). Using GPS or address data these individuals were followed up by field teams, and paired samples of 1–3 mL of blood were collected twice with a 3-month interval.

Microbiological culture of blood was done using an automated system (BD BACTEC Blood Culture System [Becton-Dickinson, Franklin Lakes, NJ, USA] or BacT/ALERT [BioMerieux, Marcy-l'Étoile, France]) at all three sites following collection of a single aerobic bottle. For positive cultures exhibiting growth, organisms were then identified using colony morphology, Gram staining, standard biochemical tests, and specific antisera, with particular focus on the identification of *S* Typhi, *S* Paratyphi, and non-typhoidal *salmonellae*.^15^ Antimicrobial susceptibility testing was done using the disc diffusion method as per the British Society for Antimicrobial Chemotherapy^16^ or Clinical and Laboratory Standards Institute^17^ guidelines; antibiotic discs used for the tests were obtained from Oxoid (Basingstoke, UK). Further details on quality control are included in the appendix (p 2).

Anti-Vi IgG titres in plasma collected from participants were measured using the commercial VaccZyme ELISA kit (The Binding Site, Birmingham, UK) according to the manufacturer's guidelines.

### Statistical analysis

We calculated crude incidence rates by dividing the number of blood-culture-confirmed cases by the total amount of person-time observed in each age group and for the entire census population in each site; and estimated 95% binomial CIs. To account for the underestimation of typhoid fever incidence based on culture-confirmed cases, we adjusted the observed crude incidence rates of *S* Typhi for blood-culture sensitivity, the probability of receiving a blood culture diagnostic test, and the probability of health-care-seeking using a Bayesian framework that incorporated both observed STRATAA data and information from the literature. Adjustments factors were site-specific and varied by age group.^18^ We calculated median values and 95% Bayesian credible intervals for the adjusted incidence estimates (appendix p 2).

We defined seroconversion as a 2-fold increase in anti-Vi IgG titres and an absolute titre of 50 EU/mL or higher at the second timepoint to account for small variations above the lower limit of detection; the absolute titre threshold was based on baseline data from presumably unexposed participants in a human challenge model.^19^ We divided the number of participants with an increase in antibody titre that met the definition for seroconversion by the total person-time in each age group, only including participants with two blood tests done approximately 3 months apart. Person-time was determined by multiplying the number of participants in each age group by the mean time between visits (in years) for the entire cohort to give an estimate of seroincidence per 100 000 person-years of observation. We estimated 95% binomial CIs.

### Role of the funding source

The study funders had no role in study design, data collection, data analysis, data interpretation, or writing of the report.

## Results

423 618 individuals residing in 99 033 households were included in the demographic censuses, contributing 626 219 person-years of observation for febrile illness surveillance (199 634 in Blantyre, 203 614 in Kathmandu, 222 971 in Dhaka; appendix p 7).

During the 2-year period of passive surveillance, 12 082 individuals who met the fever criteria (n=3594 in Blantyre, n=2473 in Kathmandu, and n=6015 in Dhaka) were enrolled from ten health-care facilities (two in Blantyre, two in Kathmandu, and six in Dhaka). Baseline characteristics for febrile patients are described in table 1.

**Table 1:** Characteristics of enrolled participants

			**Blantyre, Malawi**			**Kathmandu, Nepal**			**Dhaka, Bangladesh**		
			*S* Typhi	*S* Typhimurium	Blood-culture negative	*S* Typhi	*S* Paratyphi A	Blood-culture negative	*S* Typhi	*S* Paratyphi A	Blood-culture negative
Enrolled participants, n			115	16	3594	150	13	2230	359	95	4931
Number of enrolled patients with available data			105	12	3594	150	13	2230	359	95	4931
Enrolment location											
	Community clinic		93/105 (89%)	11/12 (92%)	3385/3594 (94%)	148/150 (98%)	13/13 (100%)	2159/2230 (97%)	352/359 (98%)	95/95 (100%)	4894/4931 (99%)
	Hospital		12/105 (11%)	1/12 (8%)	209/3594 (6%)	2/150 (2%)	0	71/2230 (3%)	7/359 (2%)	0	37/4931 (1%)
Participant characteristics											
	Sex										
		Men	53/105 (50%)	5/12 (42%)	1791/3594 (50%)	88/150 (59%)	8/13 (62%)	1262/2230 (57%)	185/359 (52%)	56/95 (59%)	2429/4931 (49%)
		Women	52/105 (50%)	7/12 (58%)	1803/3594 (50%)	62/150 (41%)	5/13 (38%)	968/2230 (43%)	174/359 (48%)	39/95 (41%)	2502/4931 (51%)
	Age, years		9·9 (6–17)*	1·6 (1·4–2·9)†	4 (2–10)‡	12·9 (8·4–21·2)*	11·9 (8–24)	8·1 (3·7–20)	8·2 (4·8–16)*	12·4 (7·6–24·1)	12·4 (5–28)
	Body temperature, °C		38·6 (38·1–39·1)*	38·3 (37·9–38·8)	38·2 (37·7–38·8)	38·3 (37–39)*	37·6 (36·9–38·2)	37·7 (36·7–38·5)	37·8 (37–38·5)*	37·8 (36·9–38·5)	37·2 (36·5–38·2)
	Duration of fever, days		6 (3–7)*	3 (2·8–8·8)	3 (2–3)	4 (3–6)*	3 (3–5)	3 (3–5)	4 (4–7)*	5 (4–7)	4 (3–7)
	Patients admitted to hospital		8/105 (8%)	2/12 (17%)	NA	5/150 (3%)	0	NA	10/359 (3%)	1/95 (1%)	NA
	Duration of hospital stay, days		NA	NA	NA	9·5 (8–10)	NA	NA	5·5 (3–7)	3 (3–3)	NA
	Deaths		2/105 (2%)	3/12 (19%)	NA	0	0	NA	0	0	NA
	Antibiotics in previous 2 weeks		28/105 (27%)*	5/12 (42%)†	281/3594 (8%)	47/150 (31%)*	3/13 (23%)	378/2230 (17%)	123/359 (34%)*	30 (32%)	774/4931 (16%)
	Antibiotic prescribed at visit to health-care facility or hospital		82/105 (78%)*	9/12 (75%)§	2693/3594 (74%)	120/150 (80%) *	11/13 (85%)§	1276/2230 (57%)‡	337/359 (94%)*	88 (93%)	2117/4931 (43%)‡

Data are n, n/N (%), or median (IQR). No cases of blood-culture-confirmed *S* Paratyphi A were identified in Blantyre and no cases of blood-culture-confirmed *S* Typhimurium were identified in Kathmandu or Dhaka. *S* Typhi=*Salmonella enterica* serotype Typhi. *S* Paratyphi=*Salmonella enterica* serotype Paratyphi. *S* Typhimurium=*Salmonella enterica* serovar Typhimurium. NA=not available.

* *S* Typhi *vs* blood-culture negative, p<0·0001.

† *S* Typhi *vs S* Paratyphi or *S* Typhimurium, p<0·0001.

‡ *S* Paratyphi or *S* Typhimurium *vs* blood-culture negative, p<0·05.

§ *S* Typhi *vs S* Paratyphi or *S* Typhimurium, p<0·05.

Pathogenic bacteria were isolated from the blood cultures of 822 (6·8%) of 12 082 participants: 162 (4·5%) in Blantyre, 166 (6·9%) in Kathmandu, and 494 (9·2%) in Dhaka. The most common bacteria were *S* Typhi (624 [76·2%] of 822 isolates; 115 in Blantyre, 150 in Kathmandu, and 359 in Dhaka), *S* Paratyphi A (108 isolates [13·0%] of 822 isolates; 13 in Kathmandu, 95 in Dhaka), and *Salmonella enterica* serovar Typhimurium (*S* Typhimurium; 16 [1·9%] of 822 isolates in Blantyre). Blood-culture positivity rates for typhoidal pathogens (*S* Typhi and *S* Paratyphi A and B) were 3·2% in Blantyre, 6·6% in Kathmandu, and 8·2% in Dhaka. The overall contamination rate was 7·9% (322 [9·0%] in Blantyre, 56 [2·3%] in Kathmandu, and 574 [9·5%] in Dhaka).

The incidence of hospital admission for typhoid fever was higher in Blantyre (eight [7·9%] hospital admissions among 105 cases) than in Kathmandu (five hospital admissions [3·3%] among 150 cases) and Dhaka (ten hospital admissions [2·5%] among 359 cases), and deaths secondary to typhoid infection were only reported in Blantyre (two deaths [1·9%] among 105 cases). Previous antimicrobial usage and prescription of antimicrobials following blood-culture collection was significantly higher in blood-culture positive individuals than blood-culture negative individuals across all three sites (table 1).

The crude incidence of blood-culture-confirmed *S* Typhi infection was 58 cases (95% CI 48–70) per 100 000 person-years of observation in Blantyre, 74 cases (62–87) per 100 000 person-years of observation in Kathmandu, and 161 cases (145–179) per 100 000 person-years of observation in Dhaka (table 2). Age-stratified unadjusted incidence for *S* Typhi was highest among individuals aged 5–9 years at all three sites (table 2); in Dhaka, the incidence rates for individuals aged 4 and 5 years was higher than the other sites (643 cases [424–936] per 100 000 person-years of observation and 620 cases [405–908] per 100 000 person-years of observation, respectively; appendix p 11).

**Table 2:** Incidence of blood-culture-confirmed typhoid fever by site and age

	**Blantyre, Malawi**			**Kathmandu, Nepal**			**Dhaka, Bangladesh**		
	Crude incidence (95% CI)	Adjusted incidence* (95% CrI)	Incidence ratio (adjusted/observed)	Crude incidence (95% CI)	Adjusted incidence* (95% CrI)	Incidence ratio (adjusted/observed)	Crude incidence (95% CI)	Adjusted incidence* (95% CrI)	Incidence ratio (adjusted/observed)
0–4 years	83 (53–124)	632 (398–965)	7·6	72 (33–136)	764 (307–1921)	10·7	417 (337–511)	2625 (1764–4244)	6·3
5–9 years	146 (103–201)	861 (599–1203)	5·9	341 (250–455)	6713 (3085–18 730)	19·7	554 (456–666)	3228 (2276–4757)	5·8
10–14 years	88 (56–132)	602 (377–915)	6·9	191 (128–275)	3750 (1653–10 559)	19·6	268 (203–348)	1564 (1050–2384)	5·8
15–29 years	32 (20–48)	361 (219–567)	11·4	92 (71–119)	1457 (684–3918)	15·8	98 (76–124)	956 (603–1635)	9·8
≥30 years	21 (10–37)	248 (124–447)	12·0	6 (2–13)	92 (29–301)	15·0	29 (19–42)	279 (157–514)	9·7
All ages	58 (48–70)	444 (347–717)	7·7	74 (62–87)	1062 (683–1839)	14·4	161 (145–179)	1135 (898–1480)	7·0

Rates are per 100 000 person-years of observation. CrI=credible interval.

* Adjusted for blood-culture sensitivity, probability of receiving a blood culture diagnostic test, and probability of health-care seeking.

*S* Paratyphi was not identified in Blantyre; overall crude incidence of blood-culture-confirmed *S* Paratyphi A infection was 6 cases (95% CI 3–10) per 100 000 person-years of observation in Kathmandu and 42 cases (34–52) per 100 000 person-years of observation in Dhaka, with the highest incidence identified among individuals aged 5–9-years.

In Blantyre, the incidence of *Salmonella* Typhimurium (causing invasive non-typhoidal Salmonella disease) was highest among individuals aged 2 years (124 cases [45–269] per 100 000 person-years of observation; appendix p 11). *S* Typhi was isolated from children younger than 2 years at all sites (appendix p 11).

**Figure 2:**
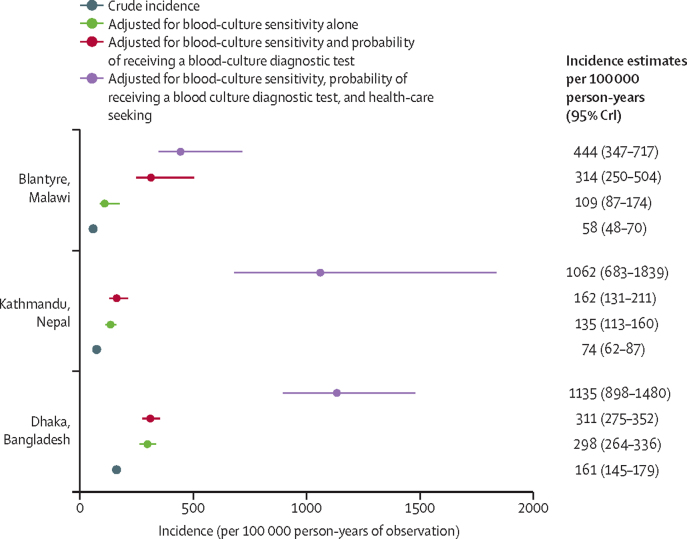
Crude observed incidence and adjusted incidence of typhoid fever caused by *Salmonella enterica* serotype Typhi across the three study sites CrI=credible interval.

The adjusted incidence of typhoid fever was highest among individuals aged 5–9-years at all three sites (table 2, figure 2). The largest adjustments at each site were for the the probability of being enrolled and receiving a blood culture in Blantyre (2·9-fold increase) and for the probability of seeking health care in Kathmandu (6·7-fold increase) and Dhaka (3·7-fold increase; table 2, figure 2).^18^

The antimicrobial susceptibility profiles for both *S* Typhi and *S* Paratyphi A differed at each site (appendix p 9). Fluoroquinolone non-susceptibility was common among *S* Typhi isolates in Dhaka (355 [99%] of 359 isolates) and Kathmandu (127 [83%] of 153 isolates), but was only identified in a single isolate from Blantyre (one [<1%] of 115 isolates). Fluoroquinolone non-susceptibility was almost universal among *S* Paratyphi A isolates (14 [100%] of 14 isolates in Kathmandu, 94 [>99%] of 95 isolates in Dhaka). In contrast, 92% of *S* Typhi isolates from Blantyre were multidrug resistant (resistant to ampicillin, chloramphenicol, and cotrimoxazole), whereas only 1% of isolates in Kathmandu and 39% of isolates in Dhaka were multidrug resistant. Azithromycin non-susceptibility was detected in both Asian sites but was not assessed in Blantyre. Azithromycin non-susceptibility was more frequent in *S* Paratyphi A (four [30%] of 14 isolates in Kathmandu, 41 [43%] of 95 isolates in Dhaka) than *S* Typhi (ten [7%] of 150 isolates in Kathmandu, 11 [3%] of 359 isolates in Dhaka).

**Figure 3:**
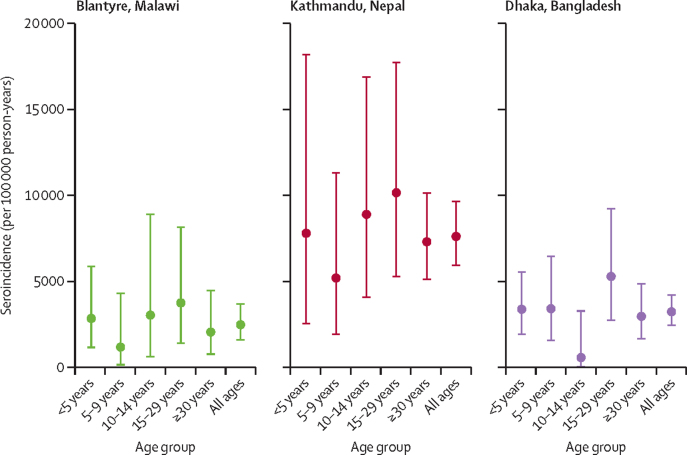
Seroincidence estimates of *Salmonella enterica* serotype Typhi exposure Seroincidence was calculated based on data from the serological survey, with randomly selected participants sampled approximately 3 months apart. Seroconversion was defined as a 2-fold rise increase in anti-Vi IgG titres and an absolute titre of 50 EU/mL or higher in the second sample at the second timepoint.

The seroconversion data showed different rates of presumed exposure to and infection with *S* Typhi in the three sites (figure 3; appendix p 15). Overall seroincidence was highest in Kathmandu (7631 episodes [95% CI 5913–9691] per 100 000 person-years of observation), was more than seven times higher than the estimated adjusted incidence, and more than 100 times higher than the observed blood-culture-confirmed incidence. The seroincidence in Blantyre (2505 episodes [1605–3727] per 100 000 person-years of observation) was five times higher than the adjusted incidence and 43 times higher than the observed incidence, and the seroincidence in Dhaka (3256 episodes [2432–4270] per 100 000 person-years of observation) was 2·5 times higher than the adjusted incidence and 20 times higher than the observed incidence (figure 3).

Similar to the incidence estimates from passive surveillance, the seroincidence analysis showed that exposure was highest among the youngest age groups (figure 3). The seroincidence estimates for Dhaka among individuals aged 0–4-years mirrored the passive surveillance estimates in which incidence was highest among individuals aged 4 years. At all sites, but particularly in Kathmandu, high rates of seroconversion were observed in the older age groups (age >30 years), in contrast with the lower age-specific incidence rates captured from passive surveillance.

## Discussion

In this STRATAA surveillance study, *S* Typhi was the primary cause of bacterial bloodstream infection in people with fever across three different epidemiological contexts in Africa and Asia. Crude incidence of blood-culture-confirmed disease identified through passive surveillance were adjusted to estimate the incidence of typhoid fever occurring within the census populations using parameters measured from within the populations, indicating that true rates are likely to be considerably higher than those estimated directly by blood culture. Seroincidence data indicate that exposure to *S* Typhi might be even higher, suggesting substantial numbers of subclinical episodes, which might make an important contribution to transmission in the population.^12^

In Dhaka, previous crude incidence estimates from population-based studies have ranged from 200 to 390 cases per 100 000 person-years of observation^20^ for all age groups, with the highest crude incidence rates (of up to 1870 per 100 000 person-years of observation) recorded in children younger than 5 years.^21^ The Global Burden of Disease Study 2017 estimated an adjusted incidence of 641 cases per 100 000 person-years of observation in Bangladesh, reduced from 1459 per 100 000 person-years of observation in 1990.^2^ In this study, the observed incidence (161 cases per 100 000 person-years of observation) and adjusted incidence (1135 per 100 000 person-years of observation) for all age groups were consistent with these previous estimates and suggest that typhoid fever incidence might not be declining as quickly as previously estimated.^2^ Adjusted incidence of *S* Typhi was as high as 3228 cases per 100 000 person-years of observation in the 5-9-year age group with the highest single-year incidence among individuals aged 4 years.

A hospital-based study from Kathmandu estimated overall crude *S* Typhi incidence to be 59 cases per 100 000 person-years of observation, averaged over a 4-year period, with a mean age of disease at 16 years.^22^ Although this estimate is similar to our observed incidence of 74 cases per 100 000 person-years of observation, our surveillance suggests that this is likely to be a substantial underestimate, with adjusted incidence of 1062 cases per 100 000 person-years of observation. In Kathmandu, the proportion of participants using alternative health-care facilities and private pharmacies was high, particularly among the 5–9-year age group, leading to a large increase in the adjusted incidence rate when compared with the crude rate. Similar to estimates for Dhaka, the highest observed and adjusted incidence was observed among the 5–9-year age group; however, these estimates also had the greatest degree of uncertainty due to the infrequent use of study facilities.

Disease incidence estimates for Africa have a greater level of uncertainty than estimates from Asia due to fewer population-based studies. A meta-analysis estimated adjusted rates of 620 cases per 100 000 person-years of observation for eastern sub-Saharan Africa and 1459 cases per 100 000 person-years of observation for central sub-Saharan Africa, although the estimates had wide CIs.^1^ In Malawi, since 2010, an increase in the number of *S* Typhi cases reported from central hospitals has been observed, with a minimum adjusted incidence estimate (accounting only for blood-culture sensitivity) of 207 cases per 100 000 person-years of observation at the peak of the typhoid fever outbreak in 2013 in Blantyre.^23^ Our results detail the ongoing high incidence of disease in Blantyre (adjusted incidence 444 cases per 100 000 person-years of observation).

The surveillance data were consistent with other published data on the increasing importance of *S* Paratyphi A in both Dhaka (117 cases per 100 000 person-years of observation) and Kathmandu (37 cases per 100 000 person-years of observation)^5^, ^24^ in the 5–9-year age group. In Blantyre, no cases of *S* Paratyphi A were identified, but consistent with surveillance across Africa,^6^ a considerable burden of *S* Typhimurium remains in the youngest children (observed incidence 117 cases per 100 000 person-years of observation among children aged 2 years).

*Salmonellae* are on the WHO priority pathogen list due to the high rate of associated antimicrobial resistance and the risk this confers to human health.^25^ Data from STRATAA surveillance are consistent with published meta-analyses on the increase in antimicrobial resistance among typhoidal *Salmonellae* and the different antimicrobial resistance profiles of *S* Typhi and *S* Paratyphi A, and differences in antimicrobial resistance between Africa and Asia.^26^ Isolates from Blantyre were almost entirely multidrug resistant, with one case of fluoroquinolone non-susceptibility documented during the surveillance period. Since STRATAA was completed, additional isolates with fluoroquinolone non-susceptibility have been detected in Blantyre (unpublished data). By contrast, in Dhaka and Kathmandu, high rates of fluoroquinolone non-susceptibility and documented azithromycin non-susceptibility were observed. These data, combined with data from passive surveillance showing individuals who were *S* Typhi culture positive had substantially higher rates of antibiotic use before seeking health care and higher rates of antibiotic prescription at the time of heath-care facility visit, demonstrate the escalating risk of antimicrobial resistance associated with global typhoid management.

Our findings demonstrate that adjustment factors cannot be applied universally to observed rates of blood-culture-confirmed disease across different epidemiological contexts and age groups. Use of alternative health-care facilities and pharmacies was higher at Asian sites than the African site, leading to higher inflation of incidence once adjusted, whereas in Blantyre, fewer individuals who sought health care at study facilities received a blood culture due to high incidence of febrile illness, inadequate health-care infrastructure, and parental refusal.^18^

In Blantyre, individuals with typhoid presented with more severe fever and a longer duration of fever, and hospitalisation was two times higher than at the other two sites. This difference might indicate that despite the adjustments for sampling and health-care seeking behaviour, undetected mild or subclinical disease remains an ongoing burden, consistent with the comparatively high rates of seroconversion observed at the Blantyre site. Such differences might also reflect differences in the immunological history of individuals across the three sites.

Measuring seroincidence, as described in this study, provides an alternative estimate of the rate of exposure to *S* Typhi than provided by passive blood-culture surveillance. A cross-sectional seroepidemiological study from Fiji estimated seroprevalence of anti-Vi IgG antibodies to be higher than predicted based on blood-culture-confirmed disease.^27^ A cross-sectional serological study in Kathmandu showed that titres of anti-Vi IgG were highest in the 16–55-year age group, consistent with our data.^28^

This study is the first to use serological data to calculate seroincidence for *S* Typhi across multiple sites. With the exception of the 10–14-year age group in Dhaka, all the age-specific seroincidence estimates either exceeded or had overlapping CIs with the adjusted incidence estimates from passive surveillance, such that seroincidence could be used as an upper bound for disease incidence within certain populations.

Quantifying the incidence of subclinical infections is also key to understanding the contribution of different age groups to the transmission of *S* Typhi. The high rates of seroconversion among older individuals, particularly in Nepal, could be evidence of ongoing exposure and transmission within these age groups, which due to the high use of antimicrobials from community pharmacies, or partial immunity leading to subclinical infection, rarely requires hospital care.

A marked difference was observed between the seroincidence estimates and the adjusted incidence estimates from passive surveillance across the three sites, with higher seroincidence rates in Kathmandu compared with Dhaka, in contrast to what was observed in passive surveillance. No established cutoff exists to define seroconversion, and anti-Vi antibody might not be the most accurate method of calculating seroincidence. Further work examining different typhoid antigens is ongoing.

This study has a number of limitations. First, due to the very large number of individuals attending health-care facilities, particularly in Blantyre, it was not possible to enrol all eligible participants. Second, due to the high number of private health-care facilities and pharmacies in both Dhaka and Kathmandu, our surveillance sites only captured a proportion of all febrile illnesses. Third, blood-culture sensitivity is only around 60% and is affected by previous antimicrobial use, volume of blood collected, and timing of blood collection in the natural history of infection. Sensitivity of blood culture is also affected by the contamination rate at collection, which was higher in Blantyre and Dhaka. Low sensitivity of blood culture is likely to result in underestimation of incidence based on blood-culture-confirmed cases, but we accounted for these factors in the adjusted estimates. Fourth, to encourage higher usage of study facilities, free treatment and care was provided within the census populations by study teams, to a variable extent at different sites. With improved diagnosis through the use of blood culture and appropriate prescription of antimicrobials, the hospital admission and complication rate might have been falsely lowered compared with other previously published observational data.^2^, ^3^, ^23^ Fifth, during the surveillance period, a proportion of the children from within these populations were enrolled into a typhoid conjugate vaccine trial, which might have introduced a degree of direct and indirect protection to the communities.^29^ Individuals who were eligible for vaccination in the trial were not included in the seroincidence analysis, which reduced the numbers of participants with paired samples, particularly in Kathmandu. The use of anti-Vi IgG seroconversion as an indicator of exposure to *S* Typhi infection is sensitive to the thresholds used to define seroconversion, which are based on scarce data. It is also possible that exposure to non-typhoidal organisms (eg, *Citrobacter*) could elicit these antibodies. Sixth, surveillance sites were chosen in urban areas with known high enteric fever incidence, and the geographical footprint and duration of surveillance was restricted. These factors together might reduce the generalisability of these data to other sites.

Despite these limitations in the design of this study, nesting multiple surveillance methods within a demographic census allowed the weaknesses of individual methods to be contextualised in different settings, and varying populations to be characterised.

Our data support the WHO recommendation for introduction of typhoid conjugate vaccines for children from age 6 months. Catch-up campaigns are also likely to be cost-effective,^30^ and should be implemented among children up to age 15 years. Our seroincidence data also suggest ongoing transmission into adulthood, which would support the use of catch-up campaigns to reduce transmission and the incidence of disease throughout the entire population. Our data support modelling work showing the impact vaccine catch-up campaigns could have on local disease incidence.^30^ The introduction of typhoid conjugate vaccines into these sites while preventing disease and reducing the high incidence identified through STRATAA, should also have an impact on overall antimicrobial use and potentially rates of resistance. High incidence of enteric fever necessitates multiple intervention strategies to achieve global control of these pathogens through development of water and sanitation infrastructure, the introduction of efficacious typhoid conjugate vaccines, and the development of vaccines for *S* Paratyphi A.

## Data sharing

Deidentified individual participant data and a data dictionary can be made available for passive surveillance, health-care utilisation, census, and census update data on request to the chief investigator (andrew.pollard@paediatrics.ox.ac.uk) with a research proposal and signed data usage agreement. A study protocol and statistical analysis plan have been published previously.^13^

## Declaration of interests

VEP is a member of the WHO Immunization and Vaccine-related Implementation Research Advisory Committee. AJP chairs the UK Department of Health Joint Committee on Vaccination and Immunisation (JCVI) and is a member of the WHO Strategic Advisory Group of Experts. RSH is supported by the National Institute for Health Research (NIHR) Global Health Research Unit on Mucosal Pathogens using UK aid from the UK Government. The views expressed in this publication are those of the authors and not necessarily those of the UK Department of Health, JCVI, WHO, NIHR, or the Department of Health and Social Care. All other authors declare no competing interests.
