# Supplementary material for: Burden of enteric fever at three urban sites in Africa and Asia: a multicentre population-based study
Source: Lancet Glob Health. 2021 Nov 16;9(12):e1688–96. doi: 10.1016/S2214-109X(21)00370-3 (PMC8609278; doi:10.1016/S2214-109X(21)00370-3)
Supplement: Supplementary appendix [file mmc1.pdf]

THE LANCET
Global Health

Supplementary appendix

This appendix formed part of the original submission and has been peer reviewed. We post it as supplied by the authors.

Supplement to: Meiring JE, Shakya M, Khanam F, et al. Burden of enteric fever at three urban sites in Africa and Asia: a multicentre population-based study. *Lancet Glob Health* 2021; **9:** e1688–96.

Appendix

Supplementary Methods

Study Sites

Malawi is a low-income country in south-eastern Africa. In Malawi, the study was conducted in Ndirande, a large urban township on the outskirts of Blantyre. Ndirande is densely populated, with limited infrastructure and poor sanitation. It is served by one health clinic and the nearby government-funded referral hospital, where a high number of *S.* Typhi cases have previously been documented.

Nepal is a lower-middle-income country in the Himalayan region of South Asia. The study was conducted in Patan, which is within the Lalitpur Sub-Metropolitan City in the Kathmandu Valley. Most people live in overcrowded conditions and obtain their water from stone spouts or sunken wells. It is served by Patan Hospital, a large government-run hospital where approximately 400 culture-confirmed cases of enteric fever are diagnosed annually.

Bangladesh is a densely populated lower-middle-income country located near the Bay of Bengal. The study was carried out in Mirpur, which is located within the capital of Dhaka and served by both the International Centre for Diarrhoeal Disease Research, Bangladesh (icddr,b) main hospital and the Mirpur Treatment Centre. Most residents have access to municipal tap water, but water quality is poor. Previous studies have found a high incidence of blood-culture-confirmed typhoid fever in Dhaka, particularly among young children.

Passive Surveillance

Participants were approached for enrolment to the passive surveillance component of the study if they had a history of fever for ≥72 hours, later changed to ≥48 hours or a recorded

temperature of >38°C. This change in recruitment was made after approximately six months of surveillance with the objective of increasing the recruitment of febrile participants and capturing as many blood-culture positive enteric fever cases within the study surveillance to accurately determine the incidence of disease.

All three sites enrolled participants of all ages from both outpatient and inpatient facilities. Surveillance was performed for two years in all sites, covering at least two complete typhoid seasons. A case of enteric fever or invasive Salmonellae infection was defined as a participant from within the demographic census area, with blood-culture confirmed Salmonellae infection (*S.* Typhi, *S.* Paratyphi A, B and C, non-typhoidal Salmonellae).

Written informed consent/assent was provided prior to enrolment. Blood was collected from participants (3-7 ml in children, 5-13 ml in adults) with priority given to aerobic blood culture bottle inoculation and the remainder collected in an EDTA tube.

<u>Quality Control at Site</u>

In Dhaka, blood cultures were performed in the Clinical Microbiology Laboratory at International Centre of Diarrhoeal Disease Research, Bangladesh (icddr,b), which operates under strict International Organization for Standardization (ISO) guidelines with ISO certification. Quality control for serological identification, ATCC strain of *S*. Typhimurium was used. E. coli ATCC 25922 and Pseudomonas aeruginosa ATCC 27853 strains were used as the quality control strains for carrying out the antimicrobial susceptibility testing.

In Kathmandu, blood culture was performed in the Microbiology Laboratory of Patan Hospital, which operates under the SOP guidelines of the hospital. For quality control, ATCC 14028 *S.* Typhimurium, ATCC 25923 Staphylococcus aureus and ATCC 25922 E. Coli were used for the growth identification, antimicrobial susceptibility and serological testing.

In Blantyre, blood cultures were performed in the Malawi Liverpool Wellcome Trust Microbiology Laboratory, which operates under its own SOP guidance.

Antimicrobial Resistance

A range of antimicrobials were tested for all isolated strains, but specifics varied depending on site and the antimicrobials available to clinical practitioners at the sites. For *S.* Typhi, multi-drug resistance (MDR) was defined as non-susceptibility to amoxicillin/ampicillin, chloramphenicol and co-trimoxazole. Bacteria found as part of the normal human skin and oral flora were defined as contaminants, including diphtheroids, bacilli, micrococci and coagulase-negative staphylococci.

Estimation of adjusted typhoid fever incidence

To estimate the adjusted typhoid fever incidence from the observed incidence of blood-culture-confirmed cases, we accounted for three steps in the observation process: (1) blood culture sensitivity, (2) enrollment and blood culture collection, and (3) healthcare seeking for (typhoid) fever. Each adjustment factor was derived from a different source. A detailed description of the Bayesian statistical approach to estimating the adjusted incidence is provided in Phillips et al.[1]

We adjusted for blood culture sensitivity using a normal mixture model based on patient-specific information about blood-culture volume and prior antibiotic use, parameterized according to the relationships with blood culture sensitivity reported in Antillon et al.[2] This resulted in a bimodal distribution for blood culture sensitivity depending on the prevalence of prior antibiotic use.

The proportion of eligible individuals who sought care but did not receive a blood culture test was directly estimated from data collected during STRATAA and subsequent local vaccine trials conducted at each site by the Typhoid Vaccine Acceleration Consortium (TyVAC).[3] For the Asian sites (Kathmandu and Dhaka), we adjusted for the higher likelihood of typhoid fever among those who had blood collected for culturing, based on a previously published imputation model that estimated that individuals who had blood drawn for culturing were 1.87 times more likely to be blood-culture positive than individuals with missing data (given data on age, duration of fever, temperature at presentation, and suspected diagnosis).[4] In Blantyre, the primary reason individuals did not have blood drawn for culturing was due to staffing shortages relative to the large number of individuals presenting to the healthcare facility, such that it was not possible to enroll and collect a blood sample from all eligible participants. Therefore, we assumed that data from individuals who did not have blood collected for culturing in Blantyre was missing compeletely at random, and the adjustment factor was estimated directly from the observed proportion of eligible individuals who were sampled.

Adjustments for healthcare seeking were based on data from the healthcare utilisation surveys and varied depending on previously identified risk factors for enteric fever.[5–7] In preliminary analyses, we found no consistent associations between severity of fever, indicators of socioeconomic status, and self-reported healthcare seeking for fever across different age groups in the three sites. Since we are interested in estimating the probability of seeking care for typhoid fever, which may differ from the probability of seeking care for fever of any cause, we used a standardization approach in which we calculated a weighted average of the probability of seeking care for fever among those with and without a previously identified typhoid risk factor.[1] For Blantyre, the risk factor was soap available after

defecation; in Nepal, it was having a household latrine; and in Bangladesh, it was boiled drinking water.[5–7] Healthcare seeking for fever was slightly higher among those with the typhoid risk factor in Malawi, but lower among those with the risk factor in Bangladesh and Nepal. To assess the robustness of our adjustments to the number of individuals sampled in the healthcare utilization survey, we also compared estimates for models that sampled the same number of individuals to models that sampled more using simulated data.

Sensitivity analysis

We additionally compared the adjusted estimates from our model to those from a simpler approach in which we assumed there was no variability in blood culture sensitivity due to prior antibiotic usage (i.e. sensitivity was normally distributed with a mean of 0.59 and standard deviation of 0.0255), there was no difference in typhoid risk between those who did or did not have blood collected for culturing (i.e. the same approach based on the enrollment fraction was used in Kathmandu and Dhaka as in Blantyre), and probability of seeking care for typhoid fever was the same as the self-reported probability of seeking care for fever in the HUS. This adjustment model and its sensitivity analyses are described in detail elsewhere.[1] Results are presented in Supplementary Table 7.

Site	Blantyre, Malawi	Kathmandu, Nepal	Dhaka, Bangladesh
Total Households from Census 1	22,364	24,405	26,119
Households with at least one child (% of total)	11,893 (53)	13,980 (57)	19,325 (74)
Households enumerated (% of total)	1,131 (5)	1478 (6)	1198 (5)

Supplementary Table 1. Sampling frame for selection of households for the healthcare utilisation surveys.

Census \ Site	Blantyre, Malawi			Kathmandu, Nepal				Dhaka, Bangladesh				
	Baseline	Final	PYO	Baseline	Update 1	Final	PYO	Baseline	Update 1	Update 2	Final	PYO
Active Households	22364	23826		24405	24897	22342		26119	26010	26281	26110	
Active Population	97392	102242	199634	101810	102590	101021	203614	110731	111418	112830	110963	222971
**Age Groups**												
0-11 months	2492	4340	6832	1202	1618	1579	2932	2120	3087	3123	2861	5596
12-23 months	2426	3844	6270	1231	1205	1166	2402	2046	2138	2180	2138	4250
24-35 months	2412	2728	5140	1200	1198	1184	2388	2071	2121	2205	2177	4288
36-47 months	2717	2655	5372	1156	1169	1168	2328	2105	2082	2102	2104	4196
48-59 months	2626	2593	5220	1265	1259	1245	2512	2150	2072	2084	2082	2095
0-4yrs	12673	16160	28833	6054	6449	6342	12563	10492	11500	11694	11362	22524
5-9yrs	12784	12576	25360	6766	6763	6701	13487	10181	10164	10251	10225	20411
10-14yrs	12991	13214	26205	7569	7665	7522	15171	10617	10665	10653	10537	21236
15-29yrs	31893	34464	66357	33380	32558	31479	62635	36097	35666	35907	35422	71546
30-49yrs	21666	20996	42662	30954	31474	31524	62634	30376	30487	30686	30593	61071
50+yrs	5385	5213	10598	17087	17430	17453	34647	12968	12936	12971	12824	25850

124 Supplementary Table 2. Demographic Census and Census Updates for the three study sites. Abbreviations: PYO, Person-years of observation

Bacteria \ Site	Blantyre, Malawi	Kathmandu, Nepal	Dhaka, Bangladesh
**Bacteria**			
E. Coli	7	1	6
Streptococcus Pneumoniae	3	0	0
Salmonella Enteritidis	1	0	0
Neisseria spp	1	0	0
Staphylococcus aureus	0	3	0
Klebsiella pneumoniae	0	2	3
Streptococcus spp	0	0	7
Eneterococcus	0	0	5
Corynebacterium spp.	0	0	3
Other	0	0	11

Supplementary Table 3. Other pathogenic non-typhoidal bacteria isolated from blood culture.

Site / Antimicrobial	Blantyre, Malawi			Kathmandu, Nepal			Dhaka, Bangladesh		
	S .Typhi			S .Typhi			S .Typhi		
**No. of cases (% of total)**	Susceptible	Non-susceptible	NT	Susceptible	Non-susceptible	NT	Susceptible	Non-susceptible	NT
Ampicillin	3 (2)	152 (98)	11	154 (99)	1 (1)	4	203 (57)	155 (43)	1
Chloramphenicol	3 (2)	163 (98)	0	149 (99)	2 (1)	8	188 (52)	167 (48)	4
Cotrimoxazole	3 (2)	163 (98)	0	150 (99)	2 (1)	7	190 (53)	168 (47)	1
MDR		152 (92)			1 (1)			140 (39)	
Amikacin	1 (100)	0 (0)	165	10 (91)	1 (9)	148	357 (99.5)	1 (0.5)	1
Amoxiclav	1 (100)	0 (0)	165	NA	NA	NA	321 (90)	35 (10)	3
Azithromycin	NA	NA	NA	140 (93)	10 (7)	9	348 (97)	11 (3)	0
Cefixime	NA	NA	NA	NA	NA	NA	359 (100)	0 (0)	0
Ceftriaxone	166 (100)	0 (0)	0	150 (100)	0 (0)	4	359 (100)	0 (0)	0
Ciprofloxacin	166 (100)	0 (0)	0	26 (17)	127 (83)	6	4 (1)	355 (99)	0
Nalidixic Acid	NA	NA	NA	19 (15)	134 (85)	6	15 (4)	344 (96)	0
Meropenem	NA	NA	NA	NA	NA	NA	356 (99.5)	0 (0.5)	3
	S. Typhimurium			S. Paratyphi			S. Paratyphi		
Ampicillin	22 (65)	12 (35)	2	14 (100)	0 (0)	0	95 (100)	0 (0)	0
Chloramphenicol	26 (72)	10 (28)	0	13 (100)	0 (0)	1	95 (100)	0 (0)	0
Cotrimoxazole	23 (64)	13 (36)	0	14 (100)	0 (0)	0	95 (100)	0 (0)	0
MDR		10 (28)							
Amikacin	NA	NA	NA	1 (100)	0 (0)	13	0 (0)	0 (0)	95
Amoxiclav	NA	NA	NA	NA	NA	NA	93 (98)	2 (2)	0
Azithromycin	NA	NA	NA	10 (70)	4 (30)	0	54 (57)	41 (43)	0
Cefixime	NA	NA	NA	NA	NA	NA	94 (100)	0 (0)	1
Ceftriaxone	36 (100)	0 (0)	0	4 (100)	0 (0)	10	95 (100)	0 (0)	0
Ciprofloxacin	27 (100)	0 (0)	0	0 (0)	14 (100)	0	1 (1)	94 (99)	0
Nalidixic Acid	NA	NA	NA	2 (15)	12 (85)		0 (0)	95 (100)	0
Meropenem	NA	NA	NA	NA	NA	NA	93 (100)	0 (0)	2

Supplementary Table 4. Antimicrobial resistance patterns for S. Typhi, S. Paratyphi A and S. Typhimurium. No cases of blood-culture confirmed *S.* Paratyphi A were identified in Blantyre and no cases of blood-culture confirmed S. Typhimurium were identified from either Kathmandu or Dhaka.

Abbreviations: NT, Not Tested; MDR, multi-drug resistance

Site	Blantyre, Malawi			Kathmandu, Nepal			Dhaka, Bangladesh		
	S. Typhi	S. Typhimurium	Blood Culture Negative	S. Typhi	S. Paratyphi	Blood Culture Negative	S. Typhi	S. Paratyphi	Blood Culture Negative
**HR (bpm)**									
<1yr	NA	115	132 (120-145)	124 (124-124)	NA	124 (115-136)	90 (89-93) *	110 (110-110)	100 (95-107)
1-2yrs	120 (120-120)	120 (87-126)	130 (115-145)	90 (90-90)	NA	120 (109-133)	98 (91-100)	85 (85-85) ^	97 (90-100)
2-5yrs	120 (106-128)	116 (106-128)	128 (115-141)	118 (108-149)	NA	120 (100-130)	92 (88-98)	92 (89-96)	92 (88-98)
5-12yrs	120 (101-122)	104 (104-104)	120 (103-130)	100 (86-108) *	124 (115-133) ^	104 (94-120)	86 (82-90)	88 (84-90)	86 (82-90)
>12yrs	105 (96-120) *	NA (86-90)	100 (88-114)	92 (84-100)	89 (86-90)	90 (80-100)	76 (74-80) *	78 (74-78)	76 (72-80)
**SBP (mmHg)**									
<1yr	NA	NA	NA	NA	NA	100 (90-140)	75 (73-80)	75 (75-75)	80 (75-85)
1-2yrs	NA	NA	NA	NA	NA	120 (109-133)	80 (75-85)	75 (75-75)	80 (72.5-87.5)
2-5yrs	NA	NA	NA	86 (86-86)	NA	120 (100-130)	85 (80-85)	85 (76-88)	85 (75-90)
5-12yrs	NA	NA	NA	90 (90-100)	90 (90-90)	100 (90-110)	90 (88-95)	90 (88-95) ^	90 (85-95) §
>12yrs	111 (103-120)	NA	117 (105-129)	100 (100-110) *	110 (105-113)	110 (100-110)	110 (100-110)	110 (100-110) ^	110 (100-120)
**MUAC (cm)**									
<1yr	NA	13 (13)	14 (13-15)	NA	NA	NA	13.6 (13.4-13.9)	13.3 (13.3-13.3)	13.7 (13-14.3)
1-4yrs	15 (14-16)	14.3 (13-16)	15 (14-16)	NA	NA	NA	14.5 (13.6-15.3)	14.3(13.4-15.2)	14.4 (13.5-15)
5-9yrs	16 (15-16.5)	17 (17)	15.5 (10-16.5)	NA	NA	NA	15.8 (14.9-17.2)	15.8 (15-17)	15.9 (14.6-
10-14yrs	18.2 (15.5-19.1)	NA	NA	NA	NA	NA	19.7 (17.4-21.8)	19.6 (17.5-20.4)	19.5 (17.9-

Supplementary Table 5. Physical characteristics of enrolled participants. Values in parentheses represent the 25th and 75th percentiles.
Abbreviations; HR, Heart Rate; bpm, beats per minute; SBP, Systolic blood pressure; mmHg, millimetres of mercury; MUAC, mid-upper arm circumference; ml, millilitres

*S. Typhi – BC Negative p-value < 0.05
^ S. Typhi – S. Paratyphi p-value <0.05
§ S. Paratyphi – BC Negative <0.05

Site / Age	Blantyre, Malawi			Kathmandu, Nepal			Dhaka, Bangladesh		
	Crude cases	Population	Unadjusted IR (95% CI)	Crude cases	Population	Unadjusted IR (95% CI)	Crude cases	Population	Unadjusted IR (95% CI)
**Age**	*S.* Typhi			*S.* Typhi			*S.* Typhi		
0-11mnths	1	3416	20 (1-112)	1	1466	34 (1-190)	3	2798	54 (11-157)
12-23mnths	1	3135	20 (1-115)	1	1201	42 (1-232)	14	2125	329 (180-553)
24-35mnths	6	2570	124 (46-271)	1	1194	41 (1-233)	24	2144	560 (359-833)
36-47mnths	6	2686	110 (41-240)	2	1164	86 (10-310)	27	2098	643 (424-936)
48-59mnths	10	2610	190 (91-350)	4	1256	159 (43-408)	26	2097	620 (405-908)
0-4yrs	24	14417	83 (53-124)	9	6282	72 (33-136)	94	11262	417 (337-511)
5-9yrs	37	12680	146 (103-201)	46	6743	341 (250-455)	113	10205	554 (456-666)
10-14yrs	23	13103	88 (56-132)	27	7585	178 (117-259)	57	10618	268 (203-348)
15-29yrs	21	33179	32 (20-48)	60	32472	92 (71-119)	70	35773	98 (76-124)
30+yrs	11	26630	21 (10-37)	6	48640	6 (2-13)	25	43460	29 (19-42)
	S. Typhimurium			*S.* Paratyphi			*S.* Paratyphi		
0-11mnths	4	3416	80 (22-205)	0	1466	0 (0-126)	0	2798	0 (0-66)
12-23mnths	6	3135	124 (45-269)	0	1201	0 (0-154)	1	2125	24 (1-131)
24-35mnths	2	2570	41 (5-150)	0	1194	0 (0-154)	4	2144	93 (25-239)
36-47mnths	1	2686	18 (0-103)	1	1164	43 (1-239)	3	2098	71 (15-209)
48-59mnths	2	2610	38 (5-138)	0	1256	40 (1-222)	4	2097	95 (26-244)
0-4yrs	15	14417	52 (29-86)	1	6282	8 (0-44)	12	11262	53 (28-93)
5-9yrs	1	12680	4 (0-22)	5	6743	37 (12-87)	24	10205	117 (75-175)
10-14yrs	0	13103	0 (0-14)	3	7585	20 (4-58)	15	10618	71 (40-117)
15-29yrs	0	33179	0 (0-6)	2	32472	3 (0-11)	33	35773	46 (32-65)
30+yrs	0	26630	0 (0-7)	2	48640	2 (0-7)	11	43460	13 (6-23)

Supplementary Table 6. Incidence Estimates. Crude cases were collected over a 24-month period, with denominator population calculated through demographic census and census update as outlined in Table2. Unadjusted incidence rates are per 100,000 person-years of observation. Abbreviations IR, Incidence rate; CI, Confidence Interval

	Blantyre, Malawi			Katrhmandu, Nepal			Dhaka, Bangladesh		
	Seroconversion Rate	95% Confidence Interval	Denominator Population	Seroconversion Rate	95% Confidence Interval	Denominator Population	Seroconversion Rate	95% Confidence Interval	Denominator Population
**Age Groups**									
0-4 years	2868	1153-5911	841	7813	2537-18232	220	3401	1904-5610	1915
5-9 years	1205	146-4352	574	5217	1915-11356	398	3435	1571-6520	1136
10-14 years	3065	631-8946	337	8910	4075-16916	348	598	15-3336	724
15-29 years	3774	1384-8213	549	10169	5255-17764	407	5309	2744-9275	980
30+ years	2076	762-4518	995	7322	5100-10183	1646	2988	1672-4928	2180
**All Ages**									
	2505	1605-3727	3296	7631	5913-9691	3023	3256	2432-4270	6935

Supplementary Table 7: Seroconversion rates calculated using anti-Vi IgG antibody from the serological survey. Visits occurred approximately 3 months apart. Seroconversion was defined as a change of two-fold rise in anti-Vi IgG plus an absolute value of 50 EU/ml in the second sample.

	Blantyre, Malawi			Kathmandu, Nepal			Dhaka, Bangladesh		
	Crude rates (95% CI)	Full model (95% CrI)	Simple approach (95% CrI)	Crude rates (95% CI)	Full model (95% CrI)	Simple approach (95% CrI)	Crude rates (95% CI)	Full model (95% CrI)	Simple approach (95% CrI)
**Age Groups**									
0-4 years	83 (53-124)	632 (398-965)	588 (370-901)	72 (33-136)	764 (307-1,921)	681 (300-1,414)	417 (337-511)	2,625 (1,764-4,244)	2,141 (1,556-3,002)
5-9 years	146 (103-201)	861 (599-1203)	807 (556-1,1143)	341 (250-455)	6,713 (3,085-18,730)	5,692 (2,950-12,963)	554 (456-666)	3,228 (2,276-4,757)	3,128 (2,317-4,296)
10-14 years	88 (56-132)	602 (377-915)	567 (353-869)	191 (128-275)	3,750 (1,653-10,559)	3,153 (1,561-7,341)	268 (203-348)	1,564 (1,050-2,384)	1,511 (1,056-2,171)
15-29 years	32 (20-48)	361 (219-567)	389 (234-614)	92 (71-119)	1,457 (684-3,918)	1,206 (648-2,541)	98 (76-124)	956 (603-1,635)	781 (541-1,145)
30+ years	21 (10-37)	248 (124-447)	266 (133-484)	6 (2-13)	92 (29-301)	76 (26-211)	29 (19-42)	279 (157-514)	227 (138-368)
**All Ages**									
	58 (48-70)	444 (347-717)	423 (339-526)	74 (62-87)	1,062 (683-1,839)	915 (661-1,308)	161 (145-179)	1,135 (898-1,480)	1,008 (830-1,232)

Supplementary Table 8: Adjusted incidence rates of typhoid fever by site and age comparing the full model to a simpler approach. Rates are per 100,000 person-years of observation. Abbreviations: CI, confidence interval; CrI, credible interval.

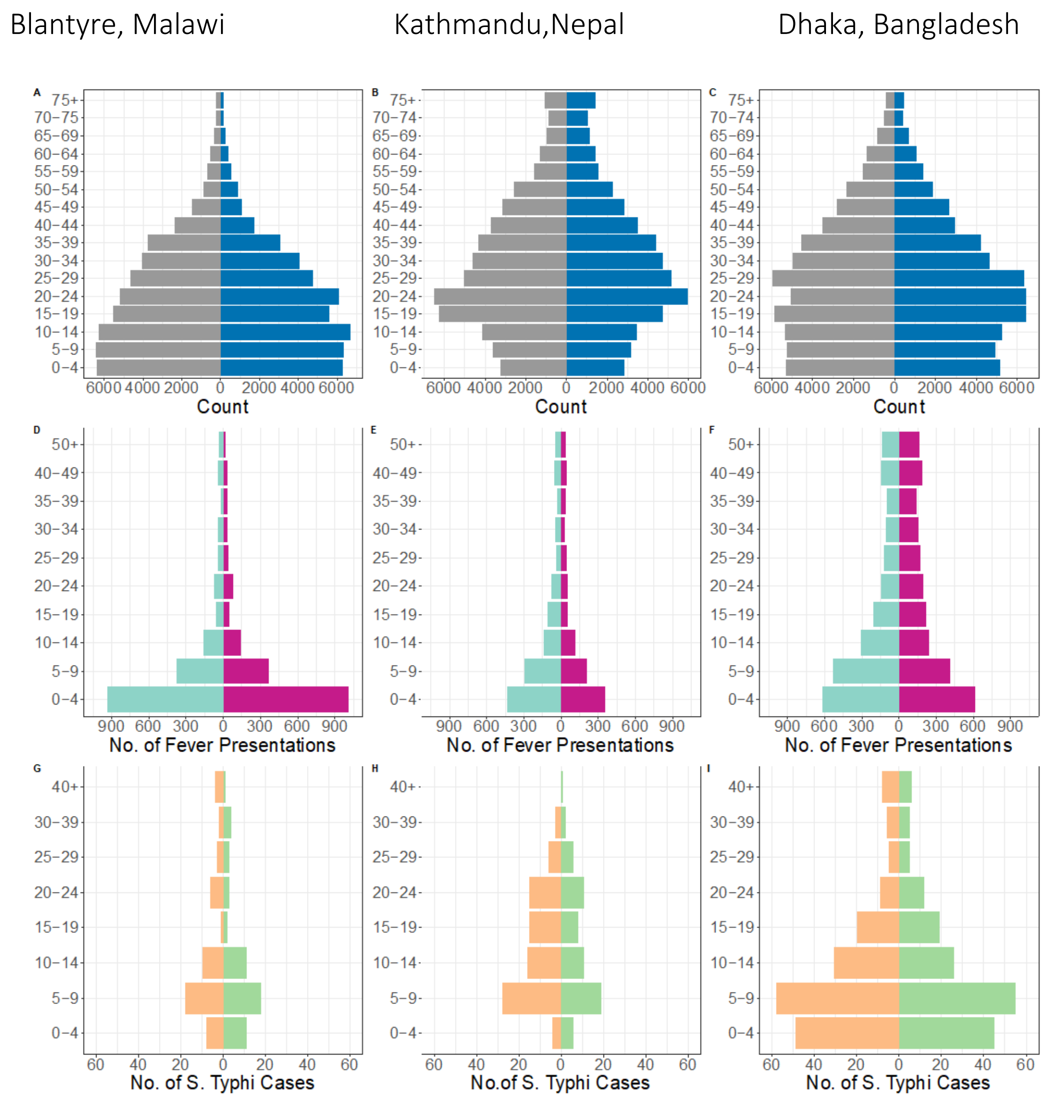

Supplementary Figure 1: Number of study participants by age and sex. (A-C) The total number of individuals enumerated in the demographic census is plotted by 5-year age group for males (grey, left) and females (blue, right). (D-F) The number of individuals presenting with fever at health facilities is plotted by age group for males (teal, left) and females (magenta, right). (G-I) Cases of blood-culture-confirmed *S.* Typhi infection is plotted by age group for males (orange, left) and females (green, right) for the three study sites.

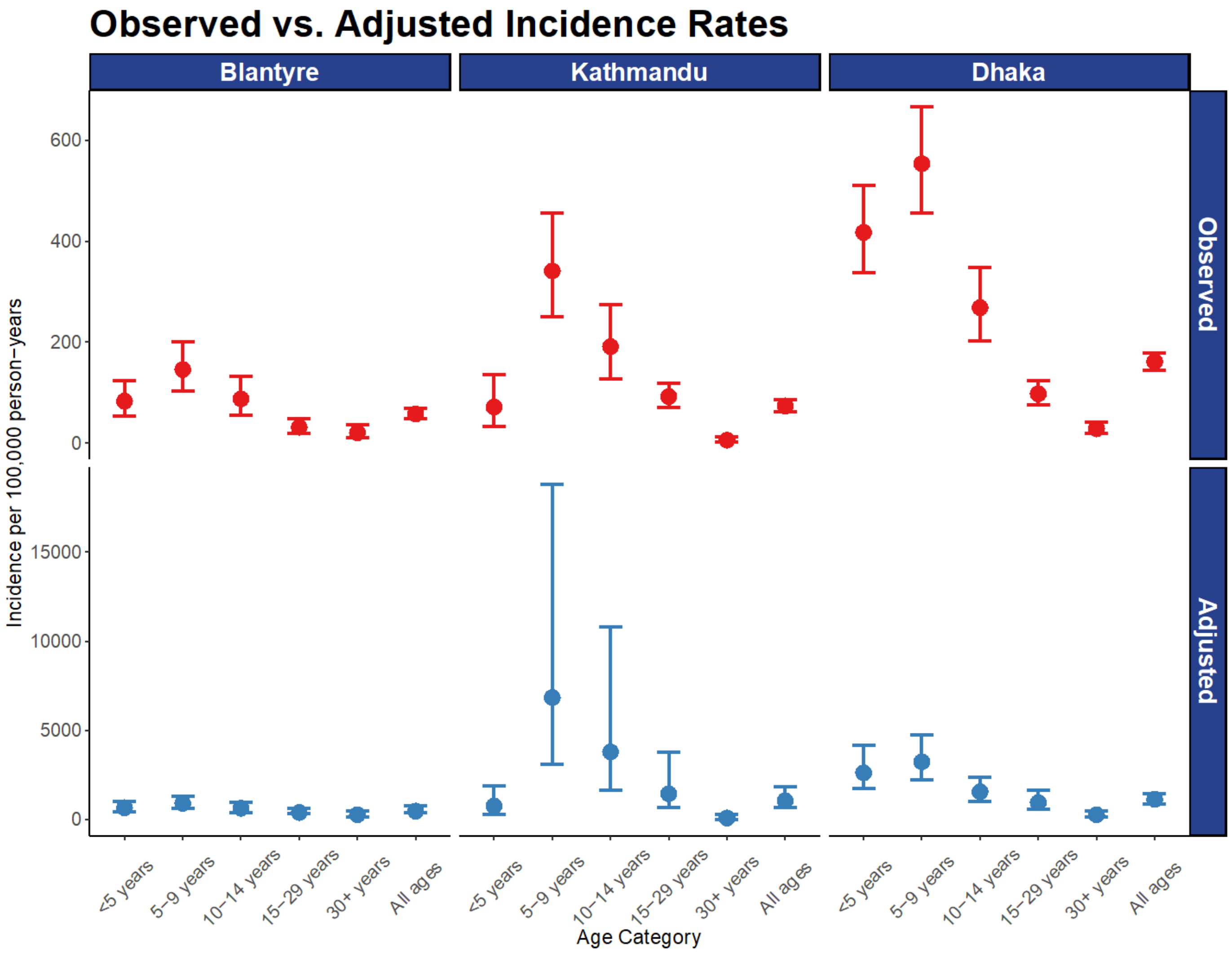

Supplementary Figure 2. Observed and adjusted incidence rates of typhoid fever across three age groups for the three study sites.

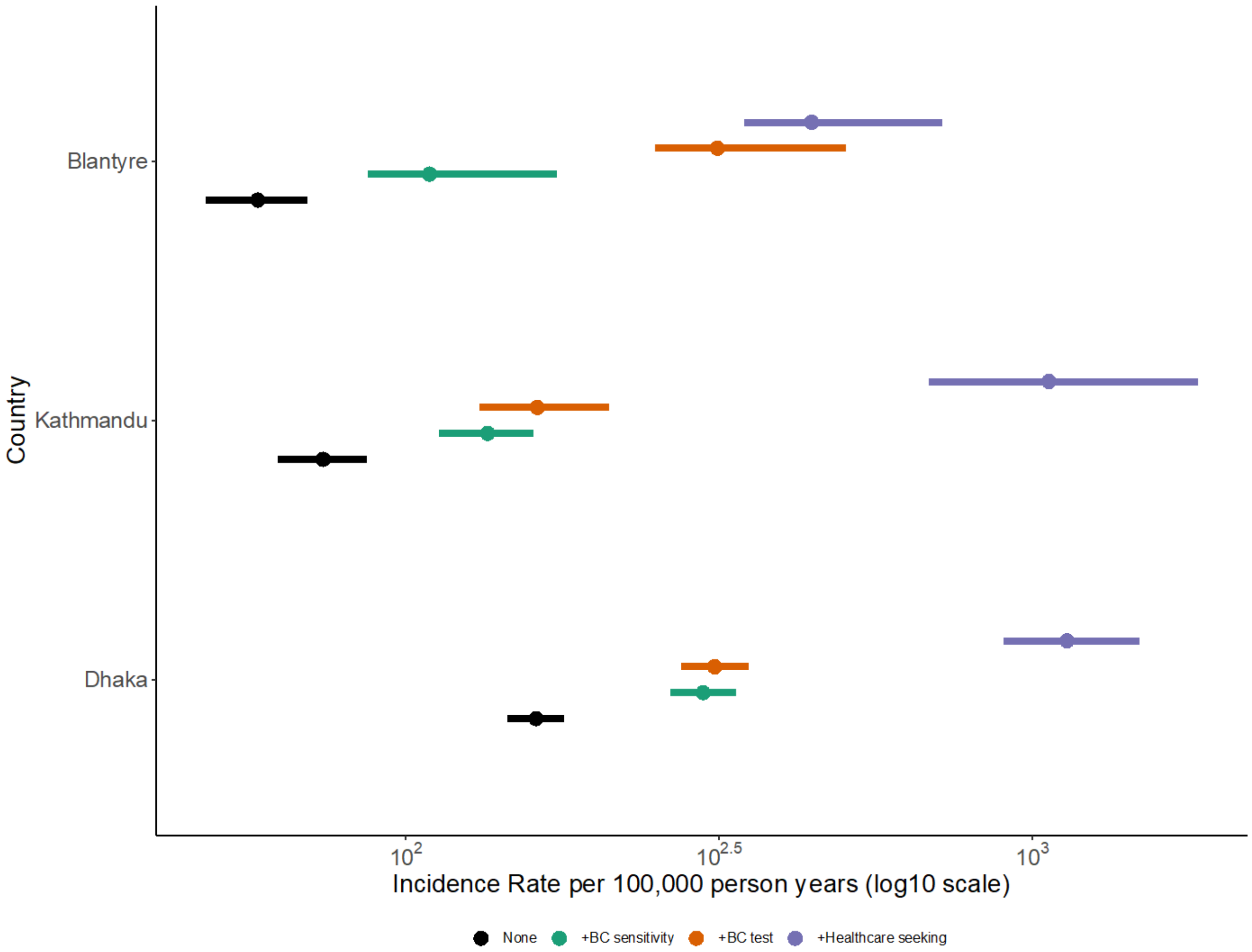

Supplementary Figure 3. Adjustments made to estimate the incidence of typhoid fever across the three study sites. The observed crude incidence of blood-culture-confirmed cases of S. Typhi (black) was adjusted incrementally for blood culture sensitivity (green), the probability of being enrolled and receiving a blood culture (orange), and probability of seeking healthcare (purple).

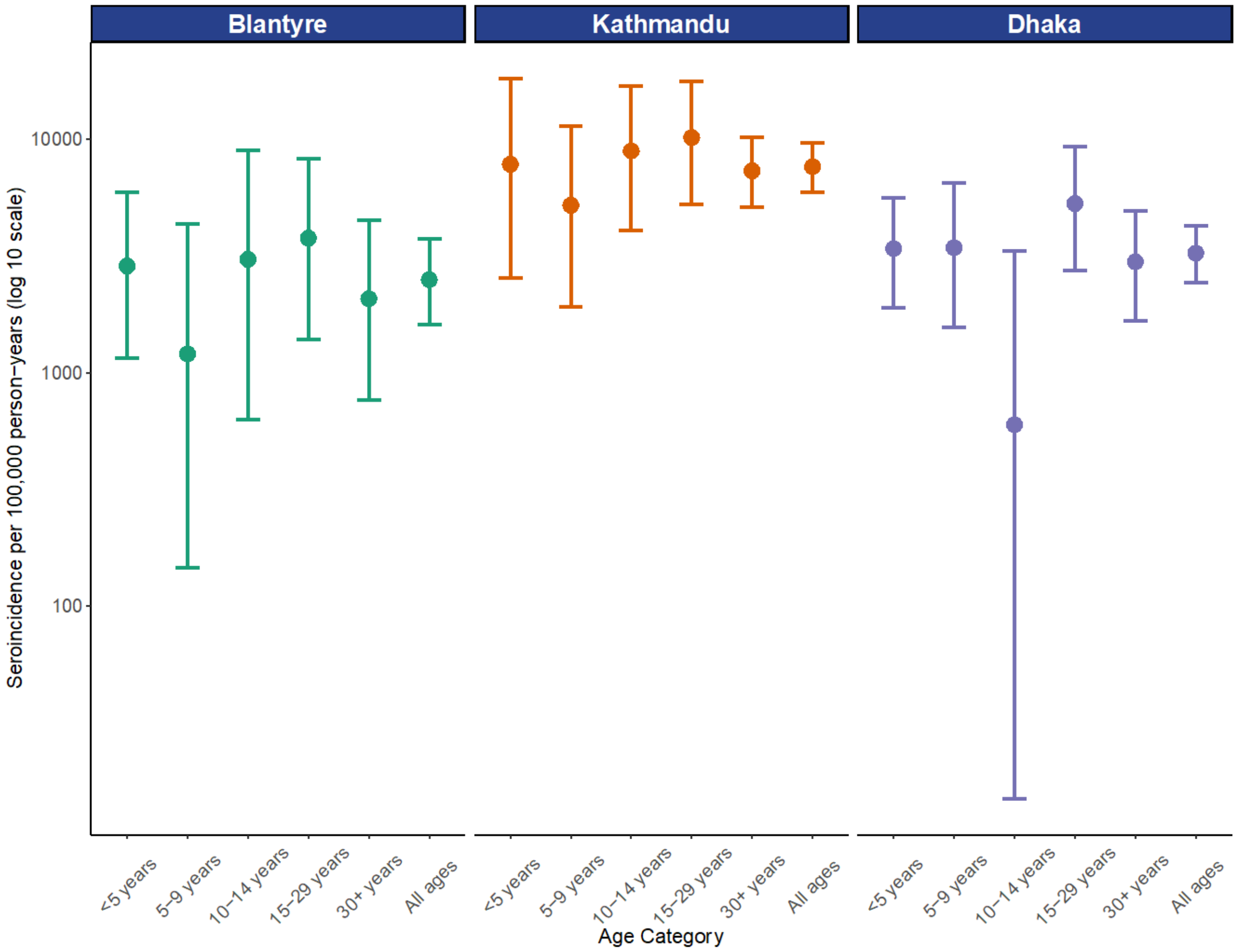

Supplementary Figure 3: Seroincidence estimates of *S.* Typhi exposure. Seroincidence was calculated based on data from the serological survey, with randomly selected participants sampled approximately 3 months apart. Seroconversion was defined as a 2-fold rise in anti-Vi IgG antibody, plus an absolute value of 50 EU/ml in the second sample.

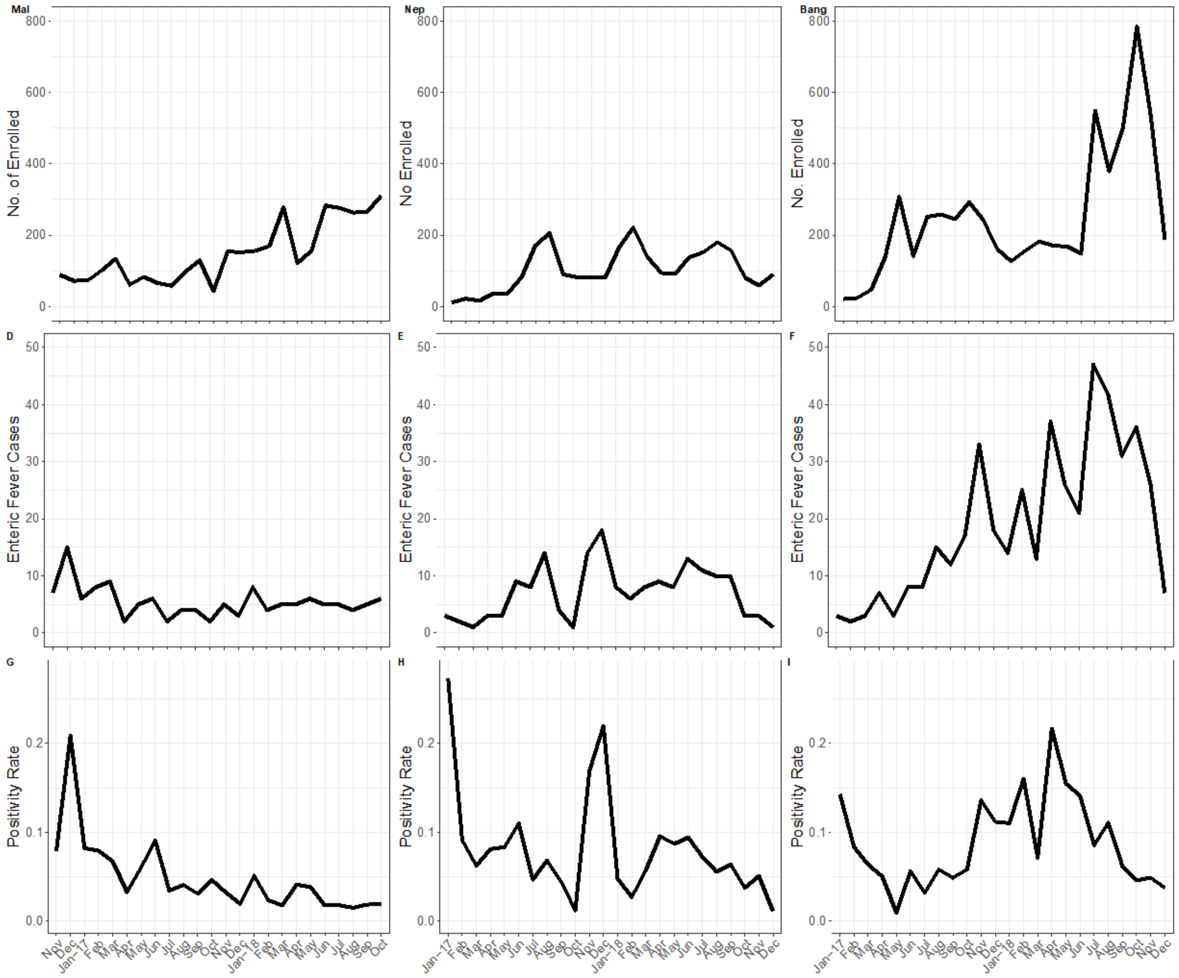

Supplementary Figure 4. Numbers of participants enrolled (row1), S. Typhi positive (row2), and positivity rate of blood cultures across a two-year period for Blantyre (column 1), Kathmandu (column 2) and Dhaka (column 3). Grey bars indicate rainy seasons across the three sites. In all three sites, recruitment of eligible individuals increased over the two-year period with an increase in recruitment as expected during the rainy seasons.

1. Phillips, M. T. *et al.* Title: A Bayesian approach for estimating typhoid fever incidence from large-scale facility-based passive surveillance data. *medRxiv* 2020.10.05.20206938 (2020). doi:10.1101/2020.10.05.20206938
2. Antillon, M., Saad, N. J., Baker, S., Pollard, A. J. & Pitzer, V. E. The Relationship Between Blood Sample Volume and Diagnostic Sensitivity of Blood Culture for Typhoid and Paratyphoid Fever: A Systematic Review and Meta-Analysis. *J. Infect. Dis.* **218**, S255–S267 (2018).
3. Meiring, J. E. *et al.* The Typhoid Vaccine Acceleration Consortium (TyVAC): Vaccine effectiveness study designs: Accelerating the introduction of typhoid conjugate vaccines and reducing the global burden of enteric fever. Report from a meeting held on 26–27 October 2016, Oxford. *Vaccine* **35**, 5081–5088 (2017).
4. Voysey, M. *et al.* Under-detection of blood culture-positive enteric fever cases: The impact of missing data and methods for adjusting incidence estimates. *PLoS Negl. Trop. Dis.* **14**, e0007805 (2020).
5. Gauld, J. S. *et al.* Domestic river water use and risk of typhoid fever: results from a case-control study in Blantyre, Malawi. *Clin. Infect. Dis.* (2019). doi:10.1093/cid/ciz405
6. Karkey, A. *et al.* Differential Epidemiology of Salmonella Typhi and Paratyphi A in Kathmandu, Nepal: A Matched Case Control Investigation in a Highly Endemic Enteric Fever Setting. *PLoS Negl. Trop. Dis.* **7**, e2391 (2013).
7. Ram, P. K. *et al.* Risk factors for typhoid fever in a slum in Dhaka, Bangladesh. *Epidemiol. Infect.* **135**, 458–65 (2007).
